# Results supporting the concept of the oxidant-mediated protein amino acid conversion, a naturally occurring protein engineering process, in human cells

**DOI:** 10.12688/f1000research.11376.2

**Published:** 2018-09-28

**Authors:** Yuichiro J. Suzuki, Jian-Jiang Hao

**Affiliations:** 1 Department of Pharmacology and Physiology, Georgetown University Medical Center, Washington, DC, 20057, USA; 2 Poochon Scientific, Frederick, MD, 21701, USA

**Keywords:** Amino acid, Glutamyl semialdehyde, Oxidative stress, Protein carbonylation, Protein engineering, Protein oxidation, Reactive oxygen species

## Abstract

Reactive oxygen species (ROS) play an important role in the development of various pathological conditions as well as aging. ROS oxidize DNA, proteins, lipids, and small molecules. Carbonylation is one mode of protein oxidation that occurs in response to the iron-catalyzed, hydrogen peroxide-dependent oxidation of amino acid side chains. Although carbonylated proteins are generally believed to be eliminated through degradation, we previously discovered the protein de-carbonylation mechanism, in which the formed carbonyl groups are chemically eliminated without proteins being degraded. Major amino acid residues that are susceptible to carbonylation include proline and arginine, both of which are oxidized to become glutamyl semialdehyde, which contains a carbonyl group. The further oxidation of glutamyl semialdehyde produces glutamic acid. Thus, we hypothesize that through the ROS-mediated formation of glutamyl semialdehyde, the proline, arginine, and glutamic acid residues within the protein structure can be converted to each other. Mass spectrometry provided results supporting that proline 45 (a well-conserved residue within the catalytic sequence) of the peroxiredoxin 6 molecule may be converted into glutamic acid in cultured human cells, opening up a revolutionizing concept that biological oxidation elicits the naturally occurring protein engineering process.

## Introduction

Reactive oxygen species (ROS) are produced through the electron reduction of molecular oxygen and include superoxide anion radicals, hydrogen peroxide (H _2_O _2_), and hydroxyl radicals ( Freeman & Crapo, 1982; Halliwell & Gutteridge, 2007). ROS have been implicated in the pathogenesis of various diseases ( Freeman & Crapo, 1982; Halliwell & Gutteridge, 2007), as well as in the aging process ( Harman, 1956; Reeg & Grune, 2015). One electron reduction of molecular oxygen produces superoxide, which in turn reacts with each other to produce H _2_O _2_ and reduces cellular iron ions. Reduced iron donates an electron to H _2_O _2_ and produces highly reactive hydroxyl radicals. Hydroxyl radicals in turn react with virtually all biological molecules, including DNA, proteins, lipids and small molecules, damaging the biological system ( Davies, 2016; Freeman & Crapo, 1982; Halliwell & Gutteridge, 2007).

**Figure 1.**
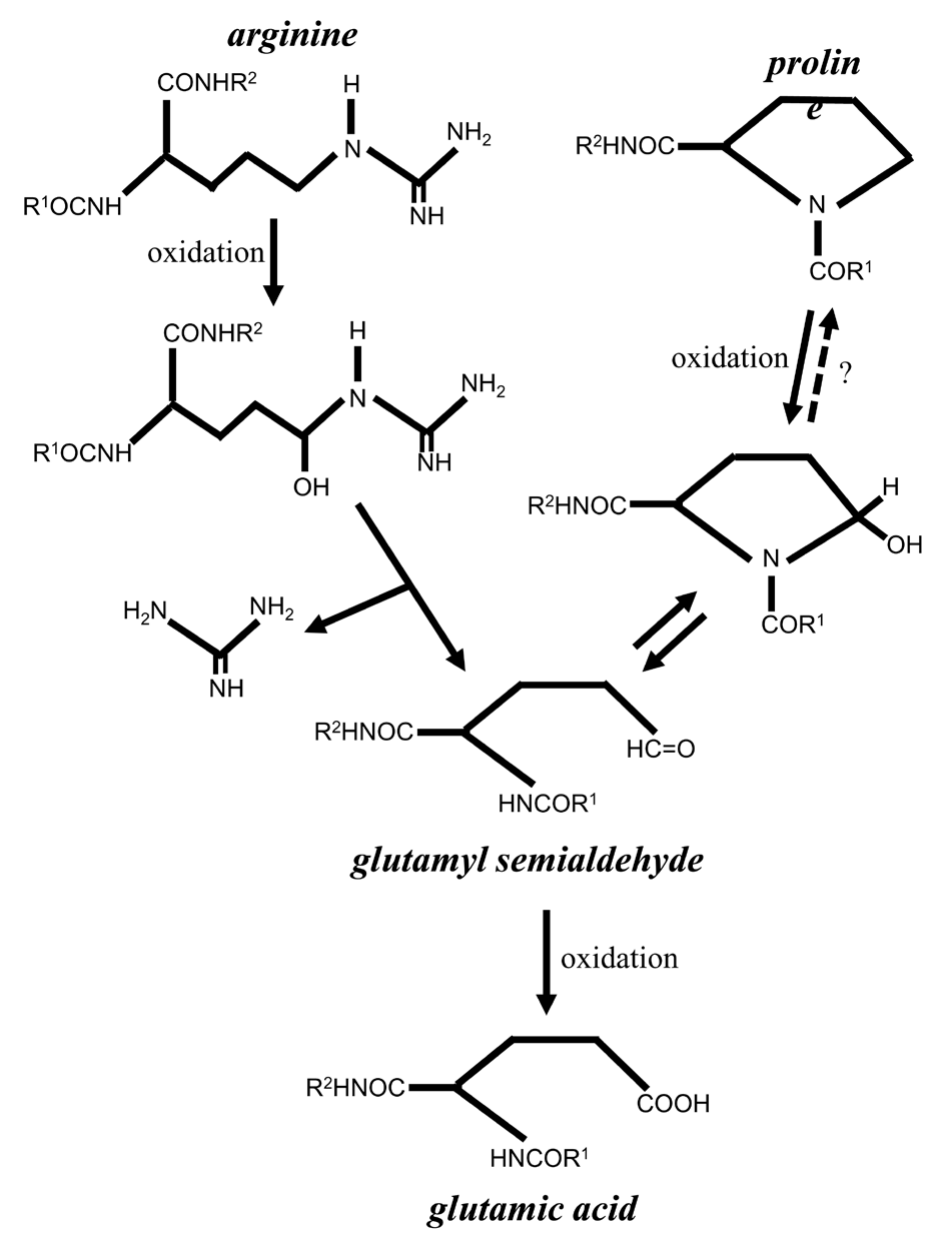
Iron-catalyzed oxidations of arginine and proline residues that result in the formation of glutamyl semialdehyde with a carbonyl group ( Amici *et al*., 1989).

One important event that occurs in response to the metal (iron)-catalyzed oxidation process is the formation of carbonyls in the protein structure. Protein carbonylation has been shown to be increased in various diseases and in aging ( Berlett & Stadtman, 1997; Levine & Stadtman, 2001; Levine, 2002; Stadtman *et al*., 1988). Protein carbonylation occurs in response to the iron-catalyzed, H _2_O _2_-dependent oxidation of amino acid side chains ( Stadtman, 1990; Suzuki *et al*., 2010). Protein carbonylation inactivates protein functions and marks damaged proteins for degradation ( Grune *et al*., 1997; Levine, 1989). While carbonylated proteins are believed not to undergo electron reduction, we previously discovered the protein de-carbonylation mechanism, in which carbonyl groups can be eliminated without proteins being degraded ( Wong *et al*., 2008). While a number of different amino acids can undergo carbonylation, major amino acid residues that are susceptible to iron-catalyzed oxidation include proline and arginine, both of which are oxidized to become glutamyl semialdehyde, which contains a carbonyl group ( Amici *et al*., 1989). Glutamyl semialdehyde is further oxidized into glutamic acid ( Figure 1).

We previously demonstrated the role of protein carbonylation in ligand/receptor-mediated cell signaling ( Wong *et al*., 2008). We further noted that the kinetics of ligand-mediated protein carbonylation is transient. Typically, in cultured cells, ligands activate the carbonylation of various proteins within 10 min and the activated protein carbonylation reverts to baseline by 30 min. These results suggest that there is a mechanism for the elimination of the formed carbonyls. We named this process “de-carbonylation” ( Wong *et al*., 2008). To understand the mechanism of de-carbonylation, we tested the hypothesis that protein carbonyls may be reduced. We found that the addition of reductants to rat heart homogenates resulted in a decrease in the protein carbonyl content ( Wong *et al*., 2013). By contrast, reductants had no effect on the carbonyl content in purified proteins, suggesting that protein carbonyls are not reduced in the absence of other cellular components. From these results, we hypothesized that cells contain catalysts for the reduction of protein carbonyls. This hypothesis is supported by our results demonstrating that the heating of heart homogenates to inactivate cellular enzymes inhibits the decrease in protein carbonyls *in vitro,* and that knocking down glutaredoxin 1 in the cells inhibits protein de-carbonylation ( Wong *et al*., 2013). We used two-dimensional gel electrophoresis and mass spectrometry to identify proteins that can be de-carbonylated and found that peroxiredoxin 6 (Prx6) is one such protein ( Wong *et al*., 2013).

Since both arginine and proline residues can be oxidized to form glutamyl semialdehyde that can further be oxidized to form glutamic acid, we speculated that arginine, proline, and glutamic acid residues may be converted to each other in the biological system, in a process that resembles site-directed mutagenesis. This article reports experimental results that support that the proline residue 45 of the human Prx6 protein molecule can be converted into glutamic acid in cells, suggesting the possible existence of a naturally occurring site-directed mutagenesis/protein engineering-like process that may be regulated by ROS.

## Methods

### Cell culture and immunoprecipitation

Human pulmonary artery smooth muscle cells (ScienCell Research Laboratories, Carlsbad, CA, USA) grown in 10 cm dishes in accordance with the manufacturer’s instructions in Smooth Muscle Cell Growth Medium (ScienCell). Some cells were serum-starved overnight with 10 ml of 0.01% fetal bovine serum-containing Dulbecco’s Modified Eagle’s medium (Mediatech, Inc., Manassas, VA, USA) for cell signaling studies. To prepare lysates, the cells were washed with phosphate buffered saline and solubilized with 1 ml of 50 mM Hepes solution (pH 7.4) containing 1% (v/v) Triton X-100, 4 mM EDTA, 1 mM sodium fluoride, 0.1 mM sodium orthovanadate, 1 mM tetrasodium pyrophosphate, 2 mM PMSF, 10 µg/mL leupeptin, and 10 µg/mL aprotinin. Cell lysates (1 ml) were immunoprecipitated with the rabbit polyclonal anti-Prx6 antibody (Sigma-Aldrich, St. Louis, MO, USA; Catalogue # P0058; 5 µg) and SureBeads Protein G Magnetic Beads (Bio-Rad Laboratories, Hercules, CA, USA; 1 mg) for 1 h at room temperature. Immunoprecipitation using SureBeads was performed in accordance with the manufacturer’s instructions.

### Peptide sample preparation

Immunoprecipitation samples were treated with dithiothreitol for reduction, then with iodoacetamide for alkylation, and further digested with trypsin (12.5 ng/µl) followed by a C18 Zip-tip clean-up (EMD Millipore, Billerica, MA, USA). Tryptic peptide samples were reconstituted in 20 µl of 0.1% formic acid before nanospray liquid chromatography/mass spectrometry/mass spectrometry (LC/MS/MS) analysis was performed.

### Nanospray LC/MS/MS analysis

The tryptic peptides mixture from each sample was analyzed using a Thermo Scientific Q-Exactive Hybrid Quadrupole-Orbitrap Mass Spectrometer (Thermo Electron, Bremen, Germany) equipped with a Thermo Dionex UltiMate 3000 RSLCnano System (Thermo Dionex, Sunnyvale, CA, USA). Tryptic peptide samples were loaded onto a peptide trap cartridge at a flow rate of 5 μl/min. The trapped peptides were eluted onto a reversed-phase 20-cm C18 PicoFrit column (New Objective, Woburn, MA, USA) using a linear gradient of acetonitrile (3–36%) in 0.1% formic acid. The elution duration was 60 min at a flow rate of 0.3 μl/min. Eluted peptides from the PicoFrit column were ionized and sprayed into the mass spectrometer using a Nanospray Flex Ion Source ES071 (Thermo Scientific, Waltham, MA, USA) under the following settings: spray voltage 1.6 kV and capillary temperature 250°C. The Q Exactive instrument was operated in the data-dependent mode to automatically switch between full scan MS and MS/MS acquisition. Survey full scan MS spectra (m/z 300−2,000) were acquired in the Orbitrap with 70,000 resolution (m/z 200) after the accumulation of ions to a 3 × 10 ^6^ target value based on predictive AGC from the previous full scan. Dynamic exclusion was set to 20 s. The 15 most intense multiply charged ions (z ≥ 2) were sequentially isolated and fragmented in the Axial Higher Energy Collision-induced Dissociation (HCD) cell using normalized HCD collision energy at 25% with an AGC target of 1e5 and a maximum injection time of 100 ms at 17,500 resolution. Two independent MS analyses in triplicate (a total of six cell samples) were performed.

### LC/MS/MS data analysis

The raw MS files were analyzed using the Thermo Proteome Discoverer 1.4.1 platform (Thermo Scientific, Bremen, Germany) for peptide identification and protein assembly. The raw data files were searched against the human protein sequence database obtained from the NCBI website ( https://www.ncbi.nlm.nih.gov) using the Proteome Discoverer software based on the SEQUEST algorithm. The carbamidomethylation of cysteines was set as a fixed modification, and Oxidation and Deamidation Q/N-deamidated (+0.98402 Da), and Pro>Glu (+31.990 Da) were set as dynamic modifications. The minimum peptide length was specified to be five amino acids. The precursor mass tolerance was set to 15 ppm, whereas fragment mass tolerance was set to 0.05 Da. The maximum false peptide discovery rate was specified as 0.01.

## Results

### Results supporting the conversion of proline residues into glutamic acid in Prx6

**Figure 2.**
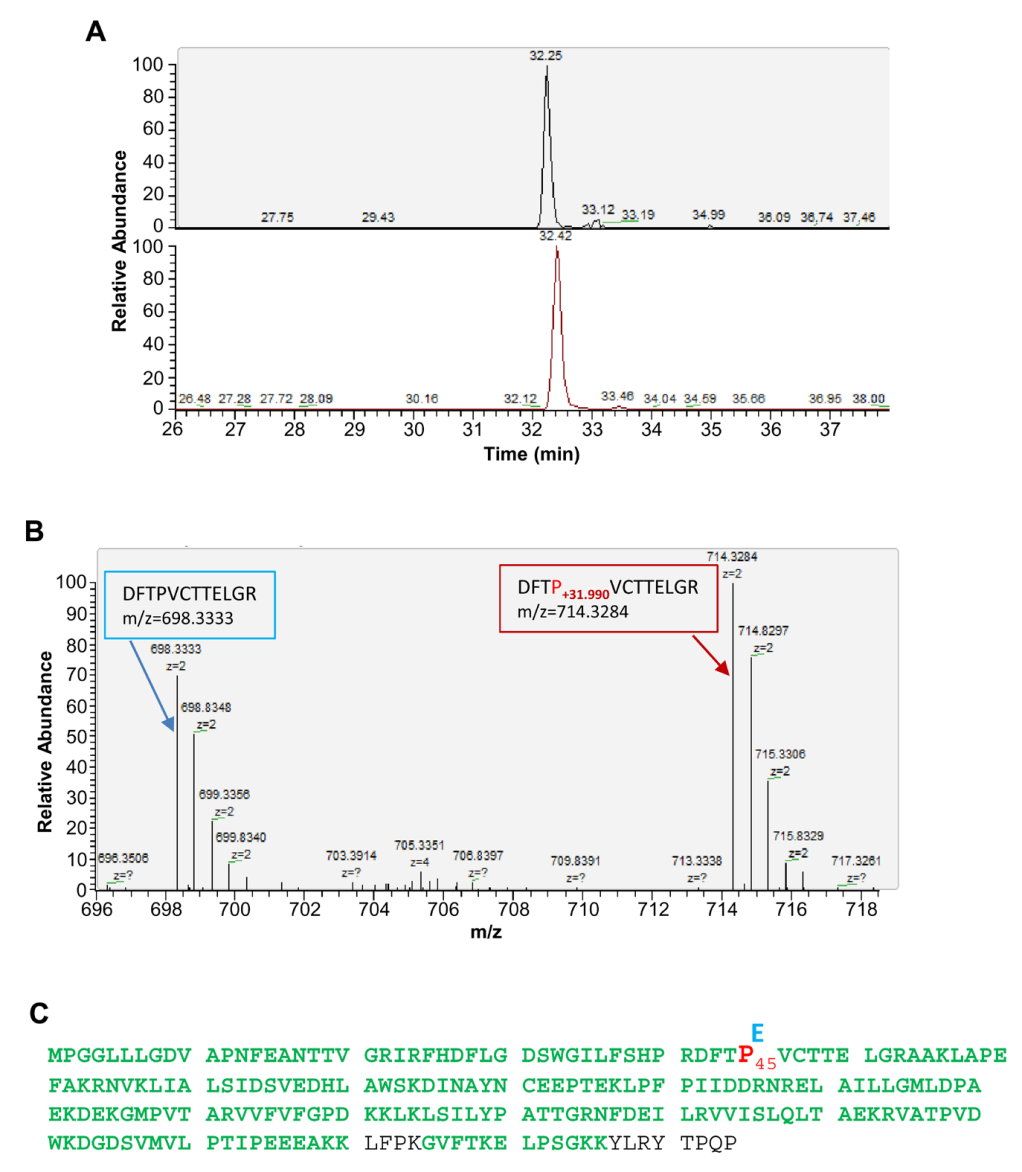
Identification of the conversion of the proline (P) residue at amino acid 45 into glutamic acid (E) in human peroxiredoxin 6 (Prx6). ( **A**) Extracted ion chromatograms of Prx6 peptide (DFT P _+31.990_ VCTTELGR, +2 charge, m/z=714.33) (top) and its non-conversion counterpart (DFTPVCTTELGR, +2 charge, m/z=698.33) (bottom). Both peptides were eluted at the same retention time and are from affinity-enriched cultured human cell extract using the anti-Prx6 antibody. ( **B**) High resolution MS spectra of the co-elution of peptides (DFTP _+31.990_ VCTTELGR, +2 charge, m/z=714.33) (right) and its non-conversion counterpart (DFTPVCTTELGR, +2 charge, m/z=698.33) (left). ( **C**) Illustration of the identified proline 45 conversion into glutamic acid in cultured human cells (shown in bold red). Sequence areas containing amino acid residues shown in green are detected by LC-MS/MS analysis after trypsin digestion.

To identify protein carbonylation sites, we enriched Prx6 by immunoprecipitation from cultured human cells. The Prx6 immunoprecipitation samples were processed for digestion by trypsin and the tryptic peptides were analyzed by nanoLC-MS/MS analysis and protein sequence alignment to identify proline sites conversion into glutamic acid in Prx6. The conversion was identified based on a mass shift of + 31.990 Da at the proline residue ( Figures 2A and B). The experiments led to the identification of one specific site at Pro 45 in human Prx6 protein ( Figure 2C).

### Confirmation of Prx6 peptides containing proline 45 modification by MS/MS

**Figure 3.**
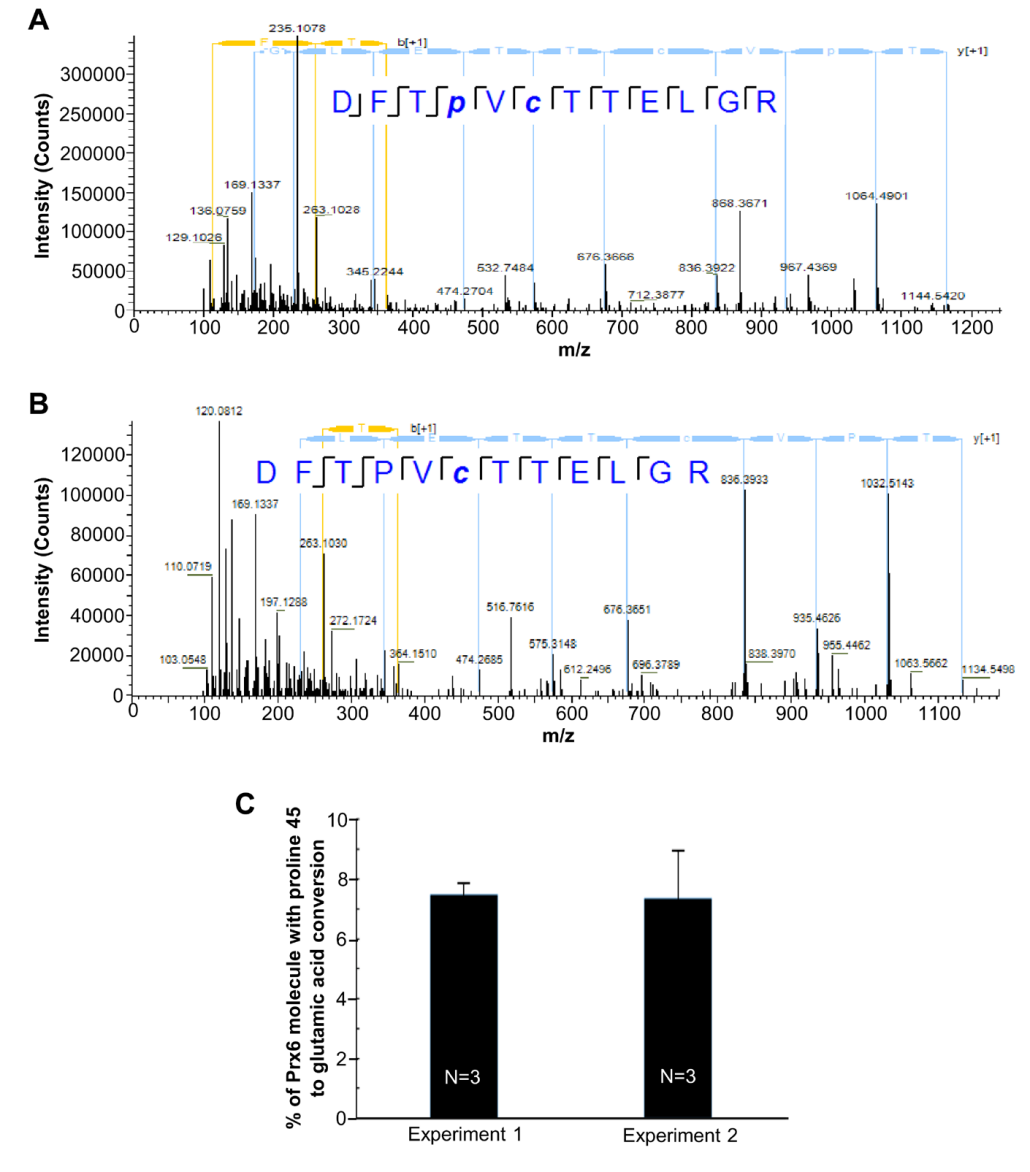
NanoLC-MS/MS verification of the conversion of proline 45 into glutamic acid at Prx6 (DFT P _+31.990_ VCTTELGR). ( **A**) High resolution MS/MS spectra of peroxiredoxin 6 (Prx6) proline to glutamic acid conversion peptide (DFT P _+31.990_ VCTTELGR). ( **B**) High resolution MS/MS spectra of Prx6 proline 45 peptide (DFTPVCTTELGR). Spectrum was obtained by LC-MS/MS analysis using the Thermo UltiMate 3000 RSLCnano System and Q Exactive Hybrid Quadrupole-Orbitrap Mass Spectrometer. ( **C**) % of Prx6 molecules with the proline 45 conversion into glutamic acid in cultured human cells. Two independent MS analyses in triplicate (a total of six cell samples) were performed.

The identified mass shift of + 31.990 Da can be caused by the conversion of proline into glutamic acid or dihydroxylated proline. Since the conversion of proline to glutamic acid or to dihydroxylated proline in Prx6 is a novel post-translational modification identified so far, it is desirable to confirm the structure of the identified peptides to ensure that the derived mass shifts of +31.99 Da are caused by the modification of proline 45. MS/MS and HPLC co-elution are gold standards for verifying peptide identification. As demonstrated in Figure 3, both peptides, DFTP+31.990VCTTELGR, +2 charge, m/z=714.33, and its non-conversion counterpart DFTPVCTTELGR, +2 charge, m/z=698.33 were co-eluted with a peak shift of less than 0.2 min. Our result showed that the high resolution MS/MS fragmentation patterns of DFTP+31.990VCTTELGR and its non-conversion counterpart DFTPVCTTELGR peptide were almost identical except the addition of +31.990 Da of fragments that contain the proline 45 residue ( Figures 3A and B).

**Figure 4.**
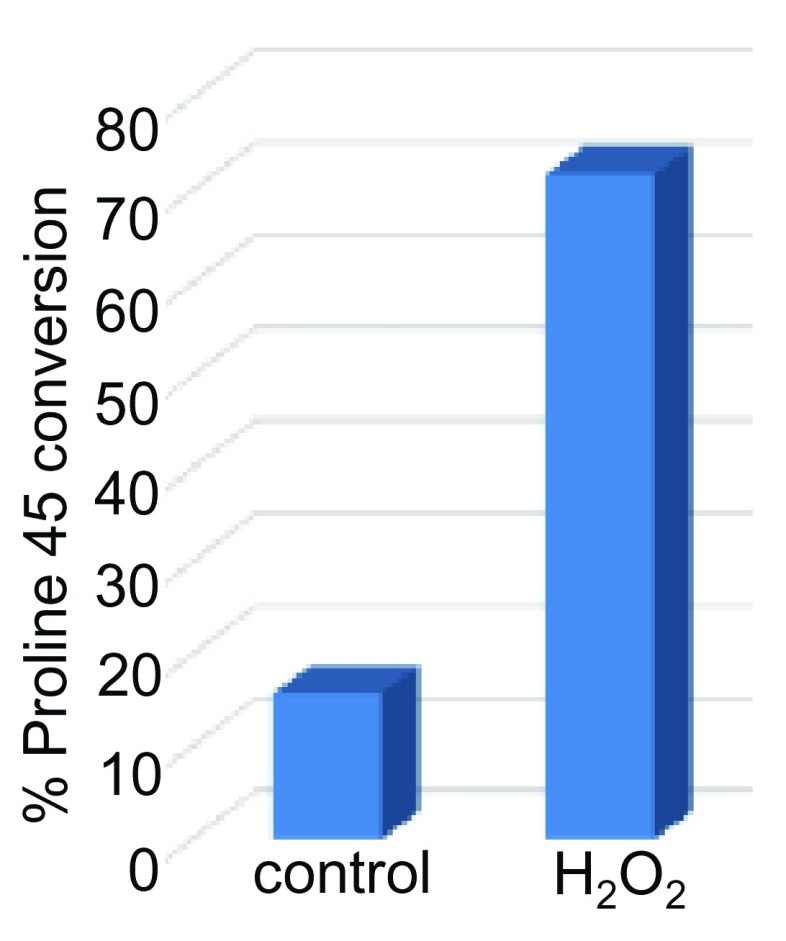
H _2_O _2_ promotes proline 45 conversion within Prx6 protein in human cells. Cells were treated with H _2_O _2_ (1 mM) for 10 min. Cell lysates were prepared and immunoprecipitated with the Prx6 antibody. Samples were subjected to MS/MS analysis. % of Prx6 with proline 45 conversion with the mass shift of + 31.990 Da increased from ~10% to ~70% after treating cells with H _2_O _2_ for 10 min.

Analysis of the ion intensity of MS spectra of DFTP+31.990VCTTELGR and its non-conversion counterpart DFTPVCTTELGR peptide ( Figure 3C) determined that the mass shift of + 31.990 Da on proline 45 occurs in 5–10% of the Prx6 molecule in our samples with a mean of 7.4 ± 1.8% (N=6). This conversion is formed post-translationally, but not due to DNA mutation, as it was promoted by treating cells with hydrogen peroxide (H _2_O _2_) for 10 min ( Figure 4). Similar results were obtained in both growing and serum-starved cells.

### The consequence of proline 45 to glutamic acid conversion in the Prx6 protein molecule

**Figure 5.**
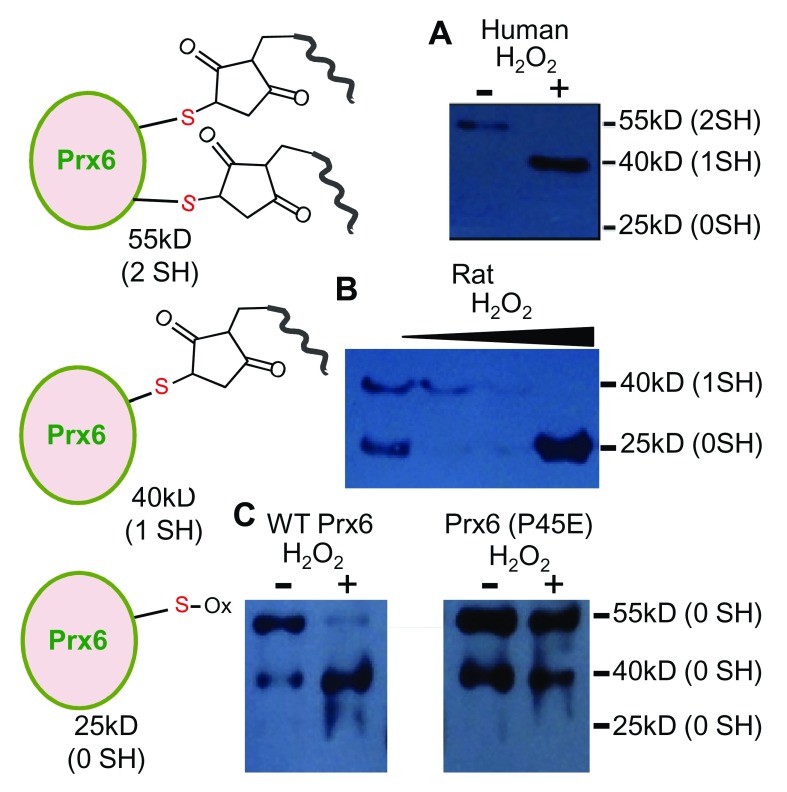
Proline 45 to glutamic acid conversion inhibits oxidation of active cysteine 47. Cultured human cells were treated with H _2_O _2_ (1 mM) for 15 min. Cellular proteins were precipitated with trichloroacetic acid and lysate samples were prepared for SulfoBiotics Protein Redox State Monitoring Kit Plus (Dojindo Molecular Technologies). Protein-SHifter that covalently binds to reduced protein thiols was added and samples were subjected to electrophoresis through a polyacrylamide gel. Each Protein SHifter causes ~15 kDa shift of the protein bands. After electrophoresis, the gel was exposed to UV irradiation to excise the Protein-SHifter Plus moiety, and then subjected to electrotransfer and Western blotting with the Prx6 antibody. In untreated cells, a 55 kDa band was primarily observed, indicating that two reduced cysteine residues in human Prx6 interacted with the SHifter. H _2_O _2_ treatment converted this to a 40 kDa band that is consistent with the Prx6 molecule with one cysteine oxidized. Human Prx6 contains two cysteine residues, one being the conserved catalytic cysteine 47 essential for its peroxidase activity and the other cysteine that is not conserved. ( **B**) Cultured rat cells were treated with H _2_O _2_ and the same experiments were performed. Rat Prx6 contains only one cysteine that is the catalytic cysteine 47. Untreated rat cell lysates exhibited the 40 kDa band that is consistent with cysteine 47 being reduced. The treatment with H _2_O _2_ caused the disappearance of the 40 kDa band, suggesting that cysteine 47 is susceptible to oxidation. ( **C**) Cells were infected with adenovirus expressing wild type (WT) Prx6 or Prx6 mutant with proline 45 replaced with glutamic acid (P45E) and treated with H _2_O _2_, followed by Protein Redox State assay. Similarly to the results in Panel **A**, ectopically expressed wild type human Prx6 exhibited a 55 kDa band without H _2_O _2_ treatment and H _2_O _2_ formed a 40 kDa band. This oxidation of cysteine 47 does not occur in mutant Prx6 (P45E), revealing that converting proline 45 to glutamic acid inhibits the oxidation of catalytic cysteine 47 by H _2_O _2_.

We found that human muscle cells treated with H _2_O _2_ exhibit oxidation of one of the two cysteine residues in human Prx6 using Dojindo SulfoBiotics Protein Redox State Monitoring Kit. In this system, 15 kDa SHifter labels free sulfhydryls and Western blotting allows for the detection of sulfhydryl oxidation in protein molecules of interest. In human Prx6 protein, one cysteine is the conserved catalytic cysteine 47 that is involved in the donation of an electron during the peroxidase activity. The other is a non-conserved cysteine, which does not occur in other species such as the rat. Untreated human cells exhibit mostly the 55 kDa band ( Figure 5A). This depicts that both of the cysteine residues are reduced in the cell and were labeled with the SHifter, resulting in a 30 kDa shift of the 25 kDa Prx6 protein. The treatment of cells with H _2_O _2_ caused the shift of this 55 kDa band to 40 kDa, suggesting that one of the two cysteines got oxidized by H _2_O _2_. To determine which cysteine may be the target of H _2_O _2_-mediated oxidation in human Prx6, rat cells in which Prx6 has only the catalytic cysteine were treated with H _2_O _2_. Figure 5B shows that untreated rat cells exhibited the 40 kDa band, suggesting that the catalytic cysteine (cysteine 47) is reduced and labeled with the SHifter. H _2_O _2_ caused the shift of this 40 kDa band to 25 kDa, indicating that cysteine 47 is the target of oxidation.

To provide information on the effects of the proline 45 to glutamic acid conversion, we constructed a human Prx6 mutant, in which proline 45 was mutated to glutamic acid, and expressed in human cells by adenovirus-mediated gene transfer. Cells expressing wild-type Prx6 and the Prx6 proline 45 to glutamic acid mutant were then treated with H _2_O _2_. Similar to the results in Figure 5A, the treatment of cells expressing wild-type human Prx6 caused the shift of the 55 kDa band to 40 kDa, indicating the oxidation of cysteine 47 by H _2_O _2_ ( Figure 5C). By contrast, this oxidation did not occur in cells expressing Pro45Glu mutant ( Figure 5C). These results indicate that the conversion of proline 45 to glutamic acid results in the inhibition of cysteine 47 oxidation by H _2_O _2_.

## Discussion

The present study introduces a revolutionizing concept that a protein engineering-like process could occur naturally in the biological system. Specifically, we provided data that may suggest that proline 45 of the Prx6 protein can be converted into glutamic acid. Proline 45 is in the peroxidase catalytic domain ( Fisher, 2011; Fisher, 2017), thus this conversion should have functional significance. Our data suggest that the modification of proline 45 indeed seems to decrease the catalytic activity of Prx6. Thus, proteins with altered amino acid sequences through oxidant-mediated conversions may confer the diversity of the functional roles of proteins in the biological system.

The results from the present study also open up a new mechanism of ROS, indicating that the protein amino acid conversion, specifically the proline–glutamic acid conversion, may be a consequence of oxidative stress mediated by the formation of glutamyl semialdehyde in the process of protein carbonylation. Through glutamyl semialdehyde, other conversions among arginine, proline, and glutamic acid are possible. Since the caged and site-directed production of hydroxyl radicals and carbonyl formation can occur via metal binding to specific sites of the protein structure ( Stadtman & Berlett, 1991; Wong *et al*., 2010), ROS-mediated protein amino acid conversion may be regulated through this mechanism. The conversion of free proline to free glutamic acid through the formation of glutamyl semialdehyde is known to occur ( Johnson & Strecker, 1962), and enzymatic mechanisms of oxidation of free glutamyl semialdehyde to free glutamic acid have been identified ( Cappelletti *et al.,* 2018). The determination of whether such mechanisms of the conversions of free amino acids also regulate protein amino acid conversions needs further investigations.

If protein amino acid conversions occur in the biological system, this would define that the DNA sequences are not the sole determinant of primary protein structures, opening up a new concept of biology.

The limitation of this study, however, is that, while the present study obtained results that are consistent with our hypothesis of oxidant-mediated protein amino acid converion, further work is needed to prove this concept.

## Data availability

The raw MS files from the output of the LC/MS/MS are available: doi, 10.17605/OSF.IO/5FN2E and 10.17605/OSF.IO/RP9J8 ( Suzuki, 2017a; Suzuki, 2017b).
