# Supplementary material for: Results supporting the concept of the oxidant-mediated protein amino acid conversion, a naturally occurring protein engineering process, in human cells
Source: F1000Res. 2018 Sep 28;6:594. Originally published 2017 Apr 28. [Version 2] doi: 10.12688/f1000research.11376.2 (PMC6192443; doi:10.12688/f1000research.11376.2)
Supplement: Peer review history

# Peer review: Referee response for version 2

**DOI:** 10.5256/f1000research.17927.r38864

**Published:** 2018-10-15

**Authors:** Dolores Pérez-Sala

**Affiliations:** 1 Biological Research Center ( CIB), Spanish National Research Council (CSIC), Madrid, Spain

In their revised version, Suzuki and Hao have addressed my concerns by expressing their conclusions more cautiously and highlighting the limitations of the study as well as the need for further work to fully confirm their hypothesis. Moreover, they have added new data that increase the interest of the study.

I have read this submission. I believe that I have an appropriate level of expertise to confirm that it is of an acceptable scientific standard.

# Peer review: Referee response for version 1

**DOI:** 10.5256/f1000research.12281.r23077

**Published:** 2017-06-28

**Authors:** Adelina Rogowska-Wrzesinska, Michael J. Davies

**Affiliations:** 1 Department of Biochemistry and Molecular Biology, VILLUM Center for Bioanalytical Sciences, University of Southern Denmark, Odense, Denmark; 2 Sydney Medical School, University of Sydney, Sydney, NSW, Australia

General overview:

 This manuscript presents an interesting aspect of the effect of ROS on proteins – the possibility of converting one type of amino acid into another one. It briefly describes the idea and presents results of a single mass spectrometry experiment that identifies two forms of a peptide obtained by trypsin digestion of Prx6 protein. One form contains proline residue and the other form contains modified form of the proline residue. The modification mass is +31.990 Da and based on that the authors conclude that oxidative stress can lead to carbonylation of proline and its further conversion to glutamic acid. No evidence is provided to proof the link between protein oxidation, ROS and the conversion of proline to glutamic acid.

 Detailed comments:

1. Abstract. Modified proteins are degraded by multiple enzymatic mechanisms not just the proteasome. Well established roles for lysosomes, LON protease and other proteases have been demonstrated.
2. The authors suggest that the Pro and Arg conversion to Glu is “interchangeable”. This statement suggest reversibility of the process, which is clearly not the case – it is a one-way reaction.
3. The conversion of one amino acid into another via oxidative reactions is definitely not a “revolutionizing concept”. It is very well established that His is converted to Asn and Asp, that Trp (and also other amino acids) can be converted to Gly (via side-chain elimination reactions), that Cys can be converted to Ala.
4. Introduction section is written with a focus on authors own work and its relevance to this work is actually not 100% clear. At the same time a lot of information is missing: Have similar processes been observed before ( *in vitro *and/or *in vivo*)? Can other amino acids undergo similar conversion processes? What is the state-of-art in this field?
5. Carbonylation is not limited to Pro and Arg ! Unfortunately this is not clear from the abstract or the introduction.
6. Methods are described in a very short form and a number of details are missing e.g. conditions for immunoprecipitation or protein digestion, amount of starting material and material used for LC-MS analysis. RAW files and processed files should be submitted to MS data repository like for example PRIDE archive.
7. It is not clear at all why the cells where starved prior the experiment and how this is linked to oxidative stress and protein carbonylation. No comparison to non-starved cells had been made.
8. It is unclear from the text that the Cys in the DFTPVCTTELGR peptide is modified. Although not stated in the methods section it seems that the samples have been reduced and alkylated because the Proteome Discoverer search parameters included carbamidomethylation of cysteines as a fixed modification.
9. +31.990 is the modification mass of proline residue. Are there any other types of post translational modifications that would result in a similar mass change?
10. Are there any other modifications present in Prx6?
11. The MS/MS spectra in figure 3 is the only evidence of the Pro to Glu conversion. Is this the only spectra that have been observed? According to methods section six samples have been analysed by LC-MS. How many times this peptide was fragmented in each sample?
  Presentation of multiple spectra would increase the credibility of the observation. Additionally the quality of the figure is not very high and it is very difficult to read the masses of the ions present in the spectra. Therefore again submitting the results to a MS data repository would help to validate the quality of the obtained results.
12. The basis of this selective oxidation is not addressed.
13. It is unclear why the authors do not test the functional significance of this modification, if they have already purified the material – it is not a very difficult assay.
14. Additional experiments where cells are collected at different conditions involving oxidative stress would help to provide the link between the carbonylation and conversion of Pro to Glu. For the moment is in not clear if this conversion is driven by oxidative stress or another unknown process.
15. The authors should not quote amino acid conversion levels based on ion intensities to 2 decimal places (7.43 +/- 1.78%). Unlikely to be this accurate.
16. The comments in the Discussion about “altered amino acids having functional roles” betrays a lack of knowledge of the protein oxidation field – this is very well established (e.g. all the work on oxidised Cys residues).
17. Many of the references cited are rather old. The most recent publications is authors own work. The field has moved on since many of these works were published.
18. The authors final statement “ROS-mediated amino acid conversion may be a tightly regulated process” should be tempered (or completely omitted). “ROS” is a very generic term, and the vast majority of oxidants do not show marked residue and site specificity. They are not “tightly regulated” in the vast majority of cases.
19. No discussion on the limitations of the presented results and conclusions is given.

We have read this submission. We believe that we have an appropriate level of expertise to state that we do not consider it to be of an acceptable scientific standard, for reasons outlined above.

# Response

**Published:** 2018-09-25

**Authors:** Yuichiro Suzuki

**Affiliations:** Department of Pharmacology, Georgetown University Medical Center, USA

**Reviewer 1**

 The paper submitted by Suzuki and Hao highlights the importance of modifications occurring in proteins as a consequence of oxidative stress. In this particular case, the authors provide data showing that proline residue at position 45 in peroxiredoxin 6 can be converted into glutamic acid residues through glutamyl semialdehyde. The results shown in the paper are well designed, solved and clear. From technical point of view, the approach is precise and gives the information necessary to draw the conclusions. Nevertheless there are some minor details that this reviewer consider that should be added or corrected in the text:

 1. The conversion of proline to glutamic semialdehyde is already known. Since the results show that the carbonyl group is further oxidized to glutamic acid, the authors should add a sentence about how they believe this last step of oxidation occurs.

 [RESPONSE: The conversion of free proline to free glutamic acid through the formation of free glutamic semialdehyde has been reported to occur. While it is not yet known whether the protein proline residue conversion to protein glutamic acid residue through the formation of glutamic semialdehyde within the protein molecule utilize the same mechanism, we have included a discussion of these published papers on free amino acids in the new version.]

 2. The authors show that P45 is transformed to E (provided a mass increase of 31,990 daltons). Did the authors check (or find) the intermediate form –the glutamic semialdehyde- and if so, to what extent this intermediate is further oxidized to glutamic acid? A brief sentence should be added to the text if they have these data.

 [RESPONSE: The reviewer makes a very important point. We did check, but in these particular samples from the present study, we only detected some proline 45 to be a structure that is consistent with glutamic acid, but not glutamic semialdehyde. Further work is needed to define the nature of protein carbonylation processes in the biological system.]

 3. There is an exciting idea concerning the concept of “naturally occurring protein engineering”. Being this true, do the authors believe that this could be a motor for evolution? Could they add a short comment on that?

 [RESPONSE: While further work is needed to prove the occurrence of protein amino acid conversion in the biological system, the present study provided data that is consistent with this concept. If it were true that posttranslational modification mechanisms can convert one type of amino acid to the other, this would imply that the DNA sequences are not the sole determinant of protein sequences. To ensure that our observations of the presumed occurrence of proline-glutamic acid conversion is not due to mutation of DNA, we treated cells with hydrogen peroxide for 10 min. This short treatment, during which gene transcription and translation processes should not be completed, caused a robust modification of proline 45, confirming that this event is post-translationally regulated. This new data has been included in Fig. 4 of the new version.]

 4. Finally, I disagree with the use of “interchangeable” in the text. This would induce to think that a protein could have a P or a E or a R in a given position without compromising its function. It is hard to believe that changing an E for an R would result in a neutral consequence. Since the consequences of such change (increase or decrease activity, stability,…) has not been proved in the case of Prx6, it seems reasonable that the term should be removed and simply say that this could be a driving force for evolution, for instance (as suggested above).

 [RESPONSE: We do mean that P, E and R can be in a given position within the protein structure. We, however, do not imply that this would not alter the function. Our theory is that such alterations would make the protein with altered functions, contributing to the diverse nature of the biological mechanisms. In fact, we performed experiments to mutate Pro45 to Glu and found that the redox interactions between peroxiredoxin 6 and hydrogen peroxide was altered. We have included this new data in Fig. 5 of the new version. We have also deleted the term “interchangeable”.]

 In the “Discussion” I would suggest the authors to change the sentence starting with “.Such studies would open up…”. I think they can really say “…studies will open up…” and change “…have functional roles…” for “can acquire new functional roles…”. Do the authors agree?

 [RESPONSE: This has been modified in the new version.]

# Response

**Published:** 2018-09-25

**Authors:** Yuichiro Suzuki

**Affiliations:** Department of Pharmacology, Georgetown University Medical Center, USA

**Reviewer 3**

 General overview:

 This manuscript presents an interesting aspect of the effect of ROS on proteins – the possibility of converting one type of amino acid into another one. It briefly describes the idea and presents results of a single mass spectrometry experiment that identifies two forms of a peptide obtained by trypsin digestion of Prx6 protein. One form contains proline residue and the other form contains modified form of the proline residue. The modification mass is +31.990 Da and based on that the authors conclude that oxidative stress can lead to carbonylation of proline and its further conversion to glutamic acid. No evidence is provided to proof the link between protein oxidation, ROS and the conversion of proline to glutamic acid.

 Detailed comments:

 Abstract. Modified proteins are degraded by multiple enzymatic mechanisms not just the proteasome. Well established roles for lysosomes, LON protease and other proteases have been demonstrated.

 [RESPONSE: In the new version, we have modified this statement in the Abstract and Introduction sections.]

 The authors suggest that the Pro and Arg conversion to Glu is “interchangeable”. This statement suggest reversibility of the process, which is clearly not the case – it is a one-way reaction.

 [RESPONSE: In the new version, we have deleted the term “interchangeable”.]

 The conversion of one amino acid into another via oxidative reactions is definitely not a “revolutionizing concept”. It is very well established that His is converted to Asn and Asp, that Trp (and also other amino acids) can be converted to Gly (via side-chain elimination reactions), that Cys can be converted to Ala.

 [RESPONSE: The reviewer is correct that conversions of free amino acids are well known, but not those of protein amino acid residues.]

 Introduction section is written with a focus on authors own work and its relevance to this work is actually not 100% clear. At the same time a lot of information is missing: Have similar processes been observed before ( *in vitro *and/or *in vivo*)? Can other amino acids undergo similar conversion processes? What is the state-of-art in this field?

 [RESPONSE: The concept of oxidant-mediated protein amino acid conversion was generated while we were studying the role of protein carbonylation in the mechanism of redox signaling. We discovered that formed protein carbonyls can be decarbonylated in the biological system, but not in purified proteins. These studies generated the concept that, in the biological system, the formation of glutamic semialdehyde from protein proline residues is reversible. This generated the idea that protein arginine residues can become protein proline residues. We initially tested this hypothesis, however, we have not yet come across this event. Instead, as described in this paper, we generated data that are consistent with the occurrence of proline-to-glutamic acid conversion that is also a process of oxidant-mediated protein amino acid conversion.]

 Carbonylation is not limited to Pro and Arg ! Unfortunately this is not clear from the abstract or the introduction.

 [RESPONSE:  In the new version, we have modified the text to make this clearer in the Introduction section.]

 Methods are described in a very short form and a number of details are missing e.g. conditions for immunoprecipitation or protein digestion, amount of starting material and material used for LC-MS analysis. RAW files and processed files should be submitted to MS data repository like for example PRIDE archive.

 [RESPONSE: In the new version, we have expanded the Methods section. The raw MS files are publicly available as described in Data availability section.]

 It is not clear at all why the cells where starved prior the experiment and how this is linked to oxidative stress and protein carbonylation. No comparison to non-starved cells had been made.

 [RESPONSE: We detected this modification in both starved cells and in non-starved cells. In the new version, we have made this statement.]

 It is unclear from the text that the Cys in the DFTPVCTTELGR peptide is modified. Although not stated in the methods section it seems that the samples have been reduced and alkylated because the Proteome Discoverer search parameters included carbamidomethylation of cysteines as a fixed modification.

 [RESPONSE: The reviewer is correct that the samples are reduced for MS analysis, thus we cannot analyze cysteine redox status using this approach. In the new version, we have added this information in the Methods section.]

 +31.990 is the modification mass of proline residue. Are there any other types of post translational modifications that would result in a similar mass change?

 [RESPONSE: As pointed by Reviewer #2, this mass shift could also be due to the formation of dihydroxylated proline. The new version of the manuscript have been modified, so that it is clear that this work presents data that are consistent with the idea of proline-to-glutamic acid conversion, but further work is needed to prove this concept.]

 Are there any other modifications present in Prx6?

 [RESPONSE: There are a number of other modifications. In the present work, we focused on providing evidence for protein amino acid conversions.]

 The MS/MS spectra in figure 3 is the only evidence of the Pro to Glu conversion. Is this the only spectra that have been observed? According to methods section six samples have been analysed by LC-MS. How many times this peptide was fragmented in each sample?

 [RESPONSE:  Experiments have been performed more than 6 times and results have been reproducible.]

 Presentation of multiple spectra would increase the credibility of the observation. Additionally the quality of the figure is not very high and it is very difficult to read the masses of the ions present in the spectra. Therefore again submitting the results to a MS data repository would help to validate the quality of the obtained results.

 [RESPONSE: The raw MS files are publicly available as described in Data availability section.]

 The basis of this selective oxidation is not addressed.

 [RESPONSE: If the concept of oxidant-mediated protein amino acid conversion is true, this defines that the DNA sequences are not the sole determinant of protein sequences, opening up a completely new concept of biology. We have stated in the new version in the Discussion section.]

 It is unclear why the authors do not test the functional significance of this modification, if they have already purified the material – it is not a very difficult assay. 

 [RESPONSE: In the new version, we have included new data showing the functional consequence of proline 45 to glutamine acid conversion in Fig. 5.]

 Additional experiments where cells are collected at different conditions involving oxidative stress would help to provide the link between the carbonylation and conversion of Pro to Glu. For the moment is in not clear if this conversion is driven by oxidative stress or another unknown process.

 [RESPONSE: In the new version, we have included new data showing that the treatment of cells with hydrogen peroxide for 10 min drives this modification in Fig. 4.]

 The authors should not quote amino acid conversion levels based on ion intensities to 2 decimal places (7.43 +/- 1.78%). Unlikely to be this accurate.

 [RESPONSE: In the new version, we have modified these.]

 The comments in the Discussion about “altered amino acids having functional roles” betrays a lack of knowledge of the protein oxidation field – this is very well established (e.g. all the work on oxidised Cys residues).

 [RESPONSE: We are strictly talking about the oxidant-mediated protein amino acid conversion process.]

 Many of the references cited are rather old. The most recent publications is authors own work. The field has moved on since many of these works were published.

 [RESPONSE: In the new version, we have added more recent references.]

 The authors final statement “ROS-mediated amino acid conversion may be a tightly regulated process” should be tempered (or completely omitted). “ROS” is a very generic term, and the vast majority of oxidants do not show marked residue and site specificity. They are not “tightly regulated” in the vast majority of cases.

 [RESPONSE: The reviewer is correct that ROS in general may not confer specificity. However, in the case of the oxidant-mediated protein amino acid conversion, such specificity may possibly regulate this process. In accordance with the reviewer’s comment, in the new version, we have deleted the term “tightly regulated”.]

 No discussion on the limitations of the presented results and conclusions is given.

 [RESPONSE: In the new version, we have added a discussion on the limitation of the present study.]

# Peer review: Referee response for version 1

**DOI:** 10.5256/f1000research.12281.r23628

**Published:** 2017-06-20

**Authors:** Dolores Pérez-Sala

**Affiliations:** 1 Biological Research Center ( CIB), Spanish National Research Council (CSIC), Madrid, Spain

In their manuscript, Suzuki and Hao report the finding of a peptide in Peroxiredoxin 6 that shows a mass increment of 32 in mass spectrometry analysis. NanoLC-MSMS analysis maps this increment at the site of a proline residue (P45 in the protein). This mass increment is found to affect approximately 7% of the peroxiredoxin 6 protein present in the samples.

 In view of these results the authors interpret that proline has suffered an oxidative modification leading to its conversion in glutamic acid.

 Our impression is that the information provided is not sufficient to establish this point.

 The mass increment of 32 Da could also be due to dihydroxylation of proline, which is a known posttranslational modification.

 Please see:

http://web.expasy.org/findmod/findmod_masses.html

 Therefore, additional experimental evidence will be required to confirm the authors’ conclusion.

 Specifically, we would suggest several of the following approaches:

 -Synthetize both the peptide with proline and with glutamic acid

 -Analyze the two peptides by HPLC. If they separate, do the same type of analysis with the peptides from their samples

 -Attempt to oxidize the proline-containing peptide in vitro (or the intact protein) to monitor the changes in proline

 -Perform amino acid analysis to confirm the presence of glutamic acid

 -Employ other derivatization or detection strategies to confirm the presence of glutamic acid.

 Ideally, the modification described could be explored in other cell types under different oxidative conditions.

 If the authors cannot obtain an unequivocal confirmation of the presence of glutamic acid, the title of the manuscript and the main interpretations should be changed.

I have read this submission. I believe that I have an appropriate level of expertise to state that I do not consider it to be of an acceptable scientific standard, for reasons outlined above.

# Response

**Published:** 2018-09-25

**Authors:** Yuichiro Suzuki

**Affiliations:** Department of Pharmacology, Georgetown University Medical Center, USA

**Reviewer 2**

 In their manuscript, Suzuki and Hao report the finding of a peptide in Peroxiredoxin 6 that shows a mass increment of 32 in mass spectrometry analysis. NanoLC-MSMS analysis maps this increment at the site of a proline residue (P45 in the protein). This mass increment is found to affect approximately 7% of the peroxiredoxin 6 protein present in the samples.

 In view of these results the authors interpret that proline has suffered an oxidative modification leading to its conversion in glutamic acid.

 Our impression is that the information provided is not sufficient to establish this point.

 The mass increment of 32 Da could also be due to dihydroxylation of proline, which is a known posttranslational modification.

 Please see:

http://web.expasy.org/findmod/findmod_masses.html

 Therefore, additional experimental evidence will be required to confirm the authors’ conclusion.

 Specifically, we would suggest several of the following approaches:

 -Synthetize both the peptide with proline and with glutamic acid

 -Analyze the two peptides by HPLC. If they separate, do the same type of analysis with the peptides from their samples

 -Attempt to oxidize the proline-containing peptide in vitro (or the intact protein) to monitor the changes in proline

 -Perform amino acid analysis to confirm the presence of glutamic acid

 -Employ other derivatization or detection strategies to confirm the presence of glutamic acid.

 [RESPONSE: We thank the reviewer for pointing out that the observed mass shift could be due to the conversion of the proline residue to dihydroxyproline. We will modified the manuscript, so that it is clear that, while we have provided data that is consistent with the idea of the proline-to-glutamic acid conversion, the present study has not proven this as it is also possible that Proline 45 is dihydroxylated. While the reviewer’s suggestion is excellent for purified proteins, our thesis is that this protein amino acid conversion is driven by biological factors, thus we need to prove this in the cell systems. We will try to find alternative avenues to prove this concept in the biological system and hope to publish such results in the future papers.]

 Ideally, the modification described could be explored in other cell types under different oxidative conditions.

 [RESPONSE: The reviewer is correct. Indeed, we are currently studying various systems including other cell types as well as tissues for patients. We hope to publish these results in the future papers.]

 If the authors cannot obtain an unequivocal confirmation of the presence of glutamic acid, the title of the manuscript and the main interpretations should be changed.

 [RESPONSE: In the new version, we have changed the title to “Results supporting the concept of the oxidant-mediated protein amino acid conversion, a naturally occurring protein engineering process, in human cells” and modified the text to make it clear that, while the present study obtained results that are consistent with our hypothesis, further work is needed to prove this concept.]

# Peer review: Referee response for version 1

**DOI:** 10.5256/f1000research.12281.r23475

**Published:** 2017-06-14

**Authors:** Joaquim Ros

**Affiliations:** 1 Department of Basic Medical Sciences, IRB-Lleida (Biomedical Research Institute of Lleida), University of Lleida, Lleida, Spain

     The paper submitted by Suzuki and Hao highlights the importance of modifications occurring in proteins as a consequence of oxidative stress. In this particular case, the authors provide data showing that proline residue at position 45 in peroxiredoxin 6 can be converted into glutamic acid residues through glutamyl semialdehyde. The results shown in the paper are well designed, solved and clear. From technical point of view, the approach is precise and gives the information necessary to draw the conclusions. Nevertheless there are some minor details that this reviewer consider that should be added or corrected in the text:

1. The conversion of proline to glutamic semialdehyde is already known. Since the results show that the carbonyl group is further oxidized to glutamic acid, the authors should add a sentence about how they believe this last step of oxidation occurs.
2. The authors show that P45 is transformed to E (provided a mass increase of 31,990 daltons). Did the authors check (or find) the intermediate form –the glutamic semialdehyde- and if so, to what extent this intermediate is further oxidized to glutamic acid? A brief sentence should be added to the text if they have these data.
3. There is an exciting idea concerning the concept of “naturally occurring protein engineering”. Being this true, do the authors believe that this could be a motor for evolution? Could they add a short comment on that?
4. Finally, I disagree with the use of “interchangeable” in the text. This would induce to think that a protein could have a P or a E or a R in a given position without compromising its function. It is hard to believe that changing an E for an R would result in a neutral consequence. Since the consequences of such change (increase or decrease activity, stability,…) has not been proved in the case of Prx6, it seems reasonable that the term should be removed and simply say that this could be a driving force for evolution, for instance (as suggested above).
5. In the “Discussion” I would suggest the authors to change the sentence starting with “.Such studies would open up…”. I think they can really say “…studies will open up…” and change “…have functional roles…” for “can acquire new functional roles…”. Do the authors agree?

I have read this submission. I believe that I have an appropriate level of expertise to confirm that it is of an acceptable scientific standard.

# Response

**Published:** 2018-09-25

**Authors:** Yuichiro Suzuki

**Affiliations:** Department of Pharmacology, Georgetown University Medical Center, USA

**Reviewer 1**

 The paper submitted by Suzuki and Hao highlights the importance of modifications occurring in proteins as a consequence of oxidative stress. In this particular case, the authors provide data showing that proline residue at position 45 in peroxiredoxin 6 can be converted into glutamic acid residues through glutamyl semialdehyde. The results shown in the paper are well designed, solved and clear. From technical point of view, the approach is precise and gives the information necessary to draw the conclusions. Nevertheless there are some minor details that this reviewer consider that should be added or corrected in the text:

 1. The conversion of proline to glutamic semialdehyde is already known. Since the results show that the carbonyl group is further oxidized to glutamic acid, the authors should add a sentence about how they believe this last step of oxidation occurs.

 [RESPONSE: The conversion of free proline to free glutamic acid through the formation of free glutamic semialdehyde has been reported to occur. While it is not yet known whether the protein proline residue conversion to protein glutamic acid residue through the formation of glutamic semialdehyde within the protein molecule utilize the same mechanism, we have included a discussion of these published papers on free amino acids in the new version.]

 2. The authors show that P45 is transformed to E (provided a mass increase of 31,990 daltons). Did the authors check (or find) the intermediate form –the glutamic semialdehyde- and if so, to what extent this intermediate is further oxidized to glutamic acid? A brief sentence should be added to the text if they have these data.

 [RESPONSE: The reviewer makes a very important point. We did check, but in these particular samples from the present study, we only detected some proline 45 to be a structure that is consistent with glutamic acid, but not glutamic semialdehyde. Further work is needed to define the nature of protein carbonylation processes in the biological system.]

 3. There is an exciting idea concerning the concept of “naturally occurring protein engineering”. Being this true, do the authors believe that this could be a motor for evolution? Could they add a short comment on that?

 [RESPONSE: While further work is needed to prove the occurrence of protein amino acid conversion in the biological system, the present study provided data that is consistent with this concept. If it were true that posttranslational modification mechanisms can convert one type of amino acid to the other, this would imply that the DNA sequences are not the sole determinant of protein sequences. To ensure that our observations of the presumed occurrence of proline-glutamic acid conversion is not due to mutation of DNA, we treated cells with hydrogen peroxide for 10 min. This short treatment, during which gene transcription and translation processes should not be completed, caused a robust modification of proline 45, confirming that this event is post-translationally regulated. This new data has been included in Fig. 4 of the new version.]

 4. Finally, I disagree with the use of “interchangeable” in the text. This would induce to think that a protein could have a P or a E or a R in a given position without compromising its function. It is hard to believe that changing an E for an R would result in a neutral consequence. Since the consequences of such change (increase or decrease activity, stability,…) has not been proved in the case of Prx6, it seems reasonable that the term should be removed and simply say that this could be a driving force for evolution, for instance (as suggested above).

 [RESPONSE: We do mean that P, E and R can be in a given position within the protein structure. We, however, do not imply that this would not alter the function. Our theory is that such alterations would make the protein with altered functions, contributing to the diverse nature of the biological mechanisms. In fact, we performed experiments to mutate Pro45 to Glu and found that the redox interactions between peroxiredoxin 6 and hydrogen peroxide was altered. We have included this new data in Fig. 5 of the new version. We have also deleted the term “interchangeable”.  ]

 In the “Discussion” I would suggest the authors to change the sentence starting with “.Such studies would open up…”. I think they can really say “…studies will open up…” and change “…have functional roles…” for “can acquire new functional roles…”. Do the authors agree?

 [RESPONSE: This has been modified in the new version.]
